# The Effects of De-Whiskering and Congenital Hypothyroidism on The Development of Nitrergic Neurons in Rat Primary Somatosensory and Motor Cortices

**DOI:** 10.22074/cellj.2018.5112

**Published:** 2018-03-18

**Authors:** Mohammad Reza Afarinesh, Gila Behzadi

**Affiliations:** 1 Sensory Processing Laboratory, Kerman Neuroscience and Cognitive Research Centers, Institute of Neuropharmachology, Kerman University of Medical Sciences, Kerman, Iran; 2 Functional Neuroanatomy Labaratory, Department of Physiology, Faculty of Medicine, Shahid Beheshti Medicine Science University, Tehran, Iran

**Keywords:** Cortex, Hypothyroidism, Nitric Oxide, Plasticity

## Abstract

**Objective:**

The aim of the present study is to investigate the effects of chronic whisker deprivation on possible alterations to the development of nitrergic neurons in the whisker part of the somatosensory (wS1) and motor (wM1) cortices in offspring with congenital hypothyroidism (CH).

**Materials and Methods:**

In the experimental study, CH was induced by adding propylthiouracil to the rats drinking water from embryonic day 16 to postnatal day (PND) 60. In whisker-deprived (WD) pups, all the whiskers were trimmed from PND 1 to 60. Nitrergic interneurons in the wS1/M1 cortices were detected by NADPH-diaphorase histochemistry staining technique in the control (Ctl), Ctl+WD, Hypo and Hypo+WD groups.

**Results:**

In both wS1 and wM1 cortices the number of nitrergic neurons was significantly reduced in the Hypo and Hypo+WD groups compared to Ctl and Ctl+WD groups, respectively (P<0.05) while bilateral whisker deprivation had no remarkable effect. The mean soma diameter size of NADPH-d labeled neurons in the Ctl+WD and Hypo+WD groups was decreased compared to the Ctl and Hypo groups, respectively. A similar patterns of decreased NADPH-d labeled neurons in the wS1/M1 cortices occur in the processes of nitrergic neurons in both congenital hypothyroidism and whisker deprivation.

**Conclusion:**

Our results suggest that both congenital hypothyroidism and whisker deprivation may disturb normal development of the wS1 and wM1 cortical circuits in which nitrergic neurons are involved.

## Introduction

Thyroid hormones (THs) regulate the normal development of cortical circuits (1). Various parts of the brain are deeply influenced during hypothyroidism. It has been well documented that congenital deficiency of THs can cause severe irreversible morphological changes in the pyramidal cortical neurons, Purkinje cells and glial cells associated with the cell hypoplasia and reduction in dendritic branching, synaptic spines, and interneuronal connections likely to cause the behavioral alterations associated with these conditions (2). In order to improve perception of the effects of thyroid dysfunction on the morphofunctional development of the brain (3), studies involving other brain areas such as somatosensory and motor cortices can be useful. In this regard, the rat vibrissal sensory system is an ideal experimental model to examine how metabolic disease factors, such as thyroid hypofunction, can influence the organization of cortical maps in developmental neuroscience (4).

In rodents, primary representation of tactile information is sent from vibrissae on the contralateral snout to the whisker part of the somatosensory cortex (wS1), known as the barrel cortex. Each barrel contains distinct cellular aggregates in layer IV of the somatosensory cortex (5). In parallel, the whisker part of the motor cortex (wM1) is an area in the agranular medial field (AGm) of the frontal cortex which controls the bilateral whisking movements (6). Principal input related to information from the whiskers in the wM1 is received predominantly via the wS1 (7).

Deprivation of peripheral sensory information inputs from whiskers, known as experience dependent plasticity, can induce cortical whisker map changes (8) that modify tactile discrimination abilities (9). Depending on the various stages of development, hypothyroidism can change the patterns of developmental processes in the brain (4). For example, a 3-5-day delay in barrel formation (revealed macroscopically by 5-HT immunostaining, succinate dehydrogenase and cytochrome oxidase histochemistry in hypothyroid rodents (10), may affect the information processing circuits.

Nicotinamide adenine dinucleotide phosphatediaphorase (NADPH-d) is a histochemical marker of nitric oxide synthase (NOS) , the enzyme responsible for the synthesis of nitric oxide (NO) (11). NADPH-d activity, as assessed by histochemistry, is used as an indirect marker for NO-producing neurons (11, 12). However, NO is a gaseous molecule associated with various essential physiological and pathological roles in the nervous system, such as acting as a transmitter and a compound of the signaling pathways that operate between blood vessels, neurons and glial cells (13). Several earlier studies have evaluated the distribution and histochemical characterization of nitrergic neurons in the brain of various mammalian species including humans (14, 15). In rats, NADPH-d neurons comprise approximately 2% of the cortical neuronal population (16). Nitrergic neurons have been observed in the wS1 (17), wM1 and the sensorimotor cortices of rodents through histochemical studies (18). The increase of cortical NADPH-d reactivity starts at postnatal day (PND) 3 and maximal activity is observed at PND 6 (19). 

Brain development is modified by various stimulations received from the environment. Some of these stimulations exert effects though sensory inputs(9), other through hormonal inputs, such as thyroidhormone (2). However, whether these stimulations independently or synergistically control brain development has not yet been clarified. The presentstudy is designed to elucidate this question in relationto the development of nitrergic neurons by combingdeprivation of sensory stimulation and TH signaling. However, the fact that TH levels regulate the activityand the level of NOS suggests crosstalk betweenTHs and the NO signaling pathway in the developingcerebral cortex of rats (20). On the other hand, removingthe whiskers of murine pups during the critical period to PND 15 leads to fused barrels and diffuseNADPH-d activity in the wS1 cortex (19). Since theeffects of chronic sensory deprivation in combinationwith congenital hypothyroidism on the developmentof nitrergic neurons have not been investigated yet inthe cortical areas of the rat, the present study aims toevaluate the morphometric characteristics of nitrergicneurons in the wS1/M1 cortical regions of congenitalhypothyroid (CH) adolescence rats following neonatal whisker deprivation to PND 60.

## Materials and Methods

In the experimental study, individual cages with a 12/12 hours light-dark cycle maintained at 22-24°C in humidity controlled conditions and with free access to food and water were supplied for four pregnant wistar rats (weighing 250-300 g). Subjects were obtained from the animal house at Shahid Beheshti University of Medical Sciences (Tehran, Iran). All of the experimental procedures were in accordance with guidelines for the care and use of laboratory animals set forth by the research council at IASP, EC/KNRC/95-9 and Shahid Beheshti University of Medical Sciences (Tehran, Iran).

Congenital hypothyroidism was induced by adding TH inhibitor, propylthiouracil (PTU, 25 mg/l, Iran hormone Co., Iran) to the drinking water of three of the pregnant dams beginning at embryonic day 16 to assure that TH levels were suppressed from the onset of fetal thyroid gland function in embryonic day (E) 17. The first day of pregnancy was determined by daily checking under sterile conditions for the first sight of a vaginal plug. Considering PND 1 as the first day of birth, PTU treatment was continued from PND 1 to PND 60 (4, 21). 

After delivery pups were placed in each cage with their mother and fresh PTU solution was prepared at weekly intervals. In this procedure the fetus and the neonates become hypothyroid as a result of the PTU treatment which reaches them through the placental barrier and after birth is transmitted to the suckling pups in the mother’s milk. TH hypofunction generally causes weight loss and feature deformity with delay in eye opening in rat offspring. This method has been proven in our laboratory to induce CH (4).

The CH induced rat dams and their pups were divided randomly into two groups as follows: In one group, known as congenitally hypothyroid whisker-deprived (Hypo+WD) offspring, all the whiskers were trimmed bilaterally every other day to a length of about 1 mm from PND 1 to PND 60 (n=4). In the other group of CH rats, the Hypo group, the whiskers of the offspring were kept intact (n=4).

A one control pregnant dam received tap water. Offspring of this pregnant dam were divided arbitrarily into two groups: the whisker deprived group (Ctl+WD), subjected to whisker trimming as explained before (n=4), and intact offspring (n=4) used as the control group (Ctl). Only male offspring were used in the present study.

### Tissue preparation

At PND 60, a mixture of ketamine (100 mg/kg, Sigma, USA) and xylazine (5 mg/kg, Sigma, USA) was utilized for anesthesia. The rats were then perfused transcardially with 0.9% saline solution, followed by 4% paraformaldehyde in phosphate-buffered saline (PBS, 0.1 M, pH=7.4) and 1. 25% picric acid. Each brain was removed from the skull and was post fixed in the same fixative overnight. The brains were then kept in 30% sucrose in PBS at 4°C for several days. They were cut coronally at 50 µm with a cryostat (Leica, USA) and collected in PBS.

### NADPH-d histochemistry

For the NADPH-d histochemistry sections were incubated in 0.2% Triton X-100 in 0.1 M PBS (pH=7.4) for 20 minutes. They were then put in a solution containing 0.5 mg/ml ß-NADPH-diaphorase (Sigma, Saint Louis, MO, USA), 0.6 mg/ml nitro blue tetrazolium (NBT, Sigma, Saint Louis, MO, USA), and 0.3% Triton X-100 dissolved in 0.1M PBS (PH=7.4) at 37°C for 1 hour. To avoid over staining, the reaction was checked every 30 minutes. Finally, sections were washed in 0.1 M PBS (PH=7.4), mounted on gelatinized glass slides, dehydrated through a series of graded alcohols, cleared in xylene, and cover slipped with Entellan.

### Morphometric analysis

**Fig.1:**
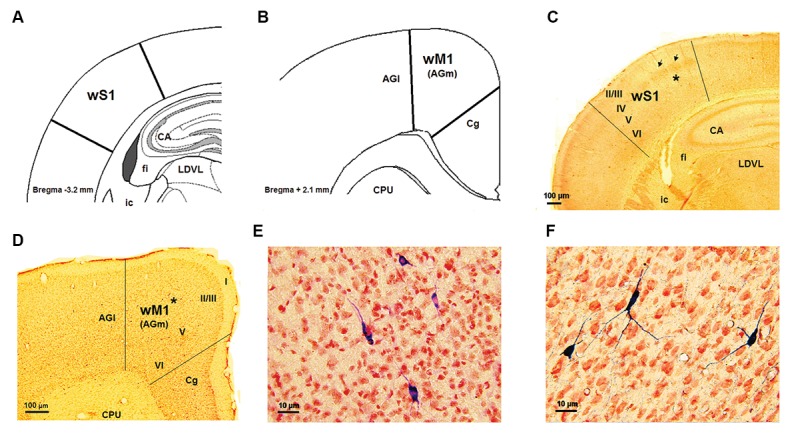
wS1/M1cortical areas and NADPH-d labeled neurons. A, B. Schematic representation sections of the wS1 and wM1 cortices, respectively, modified from the Paxinos and Watson atlas, C, D. Histograms showing coronal sections of the wS1 and wM1, respectively, E, and F. Highmagnification of some NADPH-d labeled neurons in layer V of the wS1 and wM1 cortices which is related to the asterisks places in part B and E, respectively. AGl; Agranular lateral field, AGm; Agranular medial field, CA1; Corn of amons of hippocampus, Cg; Cingulate area, CPU; Caudate putamen, ic; Internal capsule, fi; Fimberia, LDVL; Laterodorsal thalamic nucleus, ventrolateral part, wS1 and wM1; Whisker part of somatosensory and primarymotor cortices.

Type I and type II reactive neurons were identified in the rat’s barrel cortex. Type I neurons were more intensely labeled. Compared to type I neurons, type II labeled neurons had a ghost-like appearance with a small diameter cell body and dendritic trees which were poorly labeled or not labeled at all (22). Type II neurons were identified throughout the wS1 and wM1 cortical areas but these neurons could be mistaken for glial cells. However, since NADPH-d histochemistry fails adequately to reveal the dendritic trees of these cells, the present study did not evaluate type II neurons. In a coronal view, the NADPH-d neuropil reactivity provided a clear image of the layered arrangement of the wS1 cortex. Layer I displayed a band of low reaction. The diffuse histochemical product demonstrated a high reactivity between layers II and III, hence making it complicated to distinguish the limit between them. In layer IV, NADPH-d activity was heterogeneously scattered, showing barrels separated from each other by septa (less reactive regions). Layer V was defined as an area of low reactivity while enzymatic reactivity increased in layer VI, facilitating identification of the limit between this layer and the white matter (Fig .1).

Based on a previous study (6), a sharp decrease in layer IV thickness and a prominent increase in layer V thickness defined the boundary of wM1 (also called the AGm). In addition, the conspicuous boundary between the agranular lateral field (AGl) and AGm was defined by a dorsal ward expansion of layer V and decreased thickness of layer II/III in area AGl. The best recognition of the boundary between the AGm and Cingulate (Cg) areas was facilitated by the increase in thickness of layer I and layer II/III in the Cg area (Fig .1). 

For quantitative analysis of the distribution of nitrergic neurons, the wS1 and wM1 cortical areas were outlined in 8-10 homologues sections and labeled neurons, identified microscopically, were plotted inside the various laminas: II-III, IV (this layer is absent in wM1 cortex), V and VI layers. The mean numbers of NADPH-d neurons were counted within wS1 (anterior posteriori-1 to-3. 5 mm) and wM1 (anterior posteriori +1 to +3.5 mm from Bregma) cortical areas according to Paxinos and Watson atlas (23). It should be noted that the density of nitrergic neurons in layer I is not expressed in the histograms due their extreme scarcity. 

Two-dimensional reconstructions of NADPH-d positiveA neurons from wS1 and wM1 cortices were performed usingthe camera lucida system with a ×40 objective. For betterqualitative analysis of the reconstructions of NADPH-dpositive neurons, 160 (wS1) and 90 (wM1) neurons wererandomly selected from each group (n=8 hemispheres from4 animals per group). Cells were selected for reconstructionbased on the integrity of the dendritic arborization in a singlehistological section. Only cells with unequivocally completedendritic arborizations were included for analysis, meaningthat more distal dendrites were typically thin. Hence, we did not include cells whose dendrites were seemingly cut artificially or apparently had not fully reacted.

**Fig.2:**
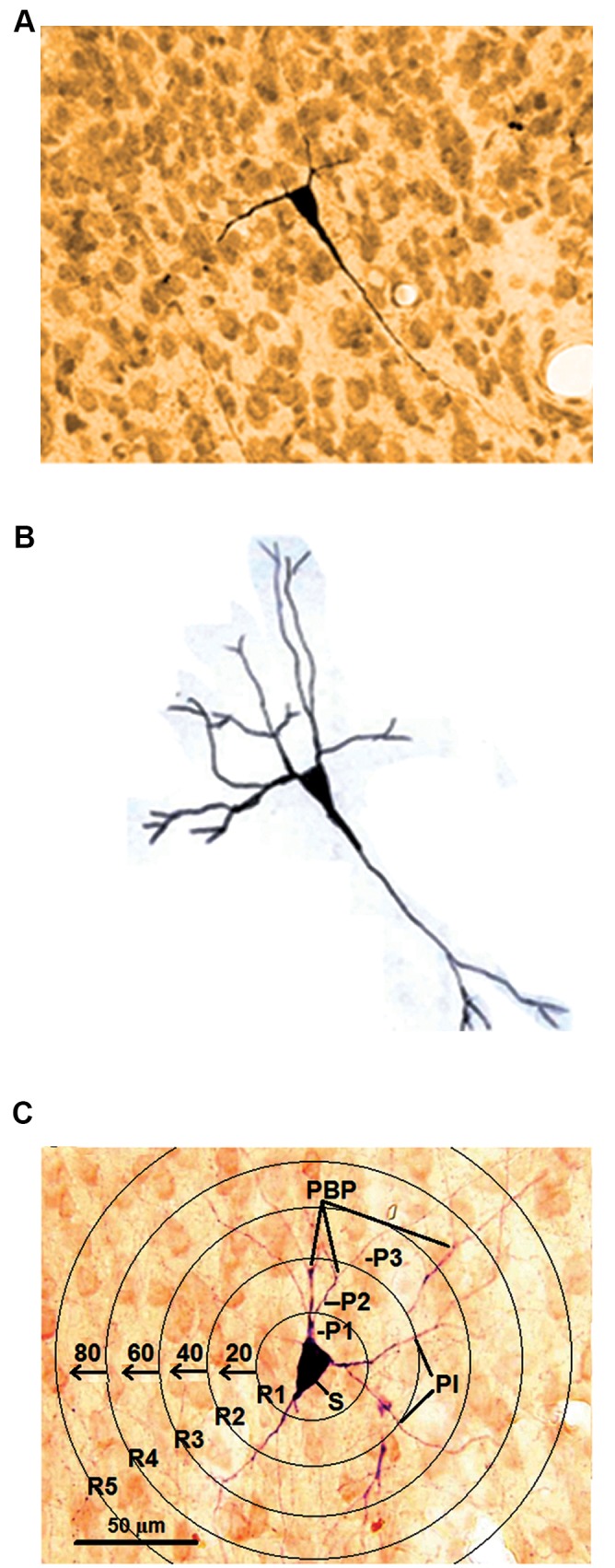
The processes quantification in a NADPH-d labeled neuron. A. Photomicrograph of a NADPH-d labeled neuron, B. Camera lucida tracing of a NADPH-d labeled neurons which is depicted in part A, and C. The processes quantification for a NADPH-d labeled neuron in the concentric rings according to Sholl analysis. R; Rings, P1-3; Processes per 1^st^, 2^nd^ and 3^rd^ orders, PB-P; Processes branching points, PI; Processes intersections, and S; Soma.

For this study, five morphometric parameters were estimated quantitatively in the suitable neurons: i. Somadiameter (measured at the maximal axis of soma), in µm (3), ii. The longest dendrites, in µm, iii. Number of processes per 1^st^, 2^nd^ and 3^rd^ orders (24), iv. Processes intersections (PIs), and v. Number of process branching points (PBPs) (25, 26).B The concentric rings (CRs) on a transparent sheet with aradial distance of 20 µm between them were used for PBPand PI quantification. The mean number of PBPs and PIs in each concentric circle were calculated (Fig .2). 

### Data analysis

Statistical differences between labeled cells in different groups were determined by the Student’s t test-student and One-Way Analysis of Variance (ANOVA) followed by Tukey’s post-hoc test (SPSS 16.00). The level of significance was set at P<0.05. 

## Results

### Body weight gain

**Fig.3:**
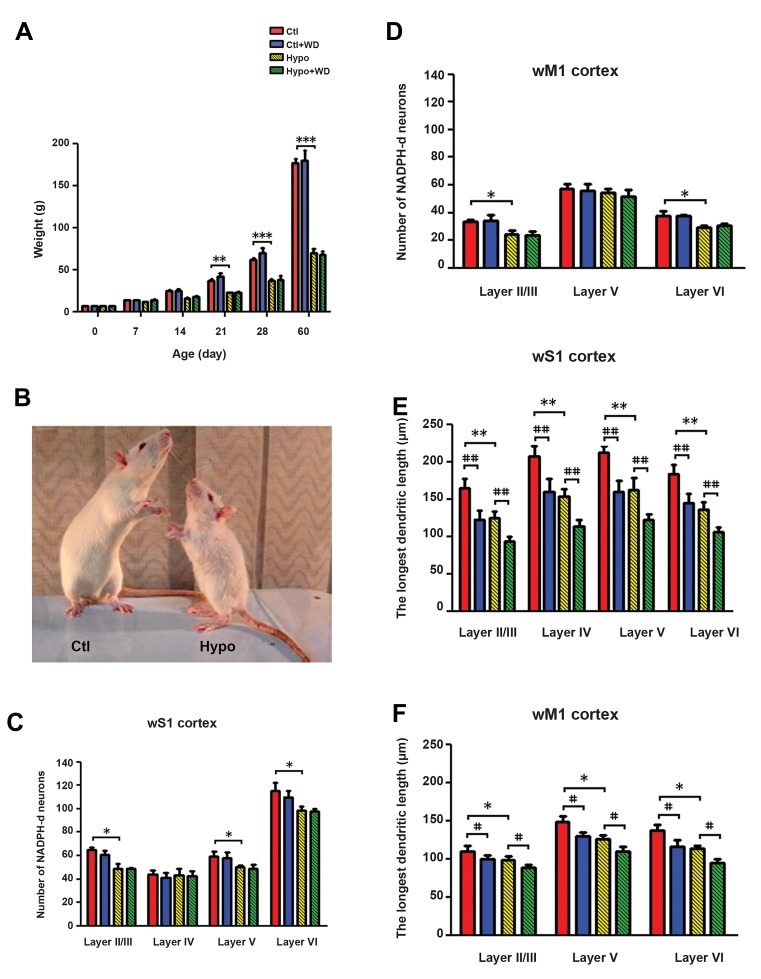
Animal’s body weight profiles in Ctl, Ctl+WD, Hypo, and Hypo+WD rats, and morphometric characteristic of NADPH-d labeled neurons of their wS1/M1 cortices. A. The body weight profile of rats from PND21 to PND60, B. Morphological deformities characteristic of hypothyroidism including: underweight, blunt snout, unfolded ears and rounded body in one case a hypothyroid rat compared to normal rat at PND 60, C, D. Number of NADPH-d neurons which was counted in laminas II/III–VI, E, and F. The longest dendrites length of NADPH-d neurons. All data are expressed as mean ± SEM. *, #; Indicate that all hypothyroid and whisker deprivation values are significantly different from the corresponding controls (*P<0.05, **P<0.01, ***P<0.001, #P<0.05, ##P<0.01), Ctl; Control, WD; Whisker-deprived, Hypo; Hypothyroid, wS1; Whisker part of somatosensory cortex, and wM1; Whisker part of primary motor cortices.

Body weight gain was reduced by 30% at PND 21 (P<0.01) and 40% at PND 28 (P<0.001), reaching 65% below normalweight gain at PND 60 (P<0.001) in the CH groups. Whiskerdeprivation had no effect on weight gain. Compared tothe normal rats, PTU-treated rats showed morphologicaldeformities characteristic of hypothyroidism, including C rounded bodies (Fig .3).

### Number and distribution

#### wS1 cortex 

In the CH (Hypo and Hypo+WD) rats, the distribution patternof NADPH-din the wS1 cortex was different from that observed in the normal (Ctl and Ctl+WD) rats. The number of NADPH-dneurons in layers II/III, V and VI was significantly decreasedin the Hypo rats compared to the Ctl group (P<0.05). Thenumber of labeled neurons in layer IV did not differ betweenthe two groups. No significant differences were observed between the numbers of NADPH-d neurons of the WD rats (Ctl+WD and Hypo+WD) and their homologues controls (Ctland Ctl+WD) respectively (Fig .3C). The total distribution ofNADPH-d positive cells was similar in the normal rats (Ctl andCtl+WD); approximately 25, 15, 20 and 40% of stained cellswere located in layers II/III, IV, V and VI of the wS1 cortex, respectively. In CH rats about 15, 20, 20 and 44% of NADPH-dpositive neurons were distributed in layers II/III, IV, V and VI(respectively) of the wS1 cortical area (Table 1).

**Table 1:** Total laminar distribution of NADPH-d neurons of the wS1 cortex and the wM1 cortex in the experimental groups


	Group			
	Ctl	Ctl+WD	Hypo	Hypo+WD
	n (%)	n (%)	n (%)	n (%)

wS1 cortex				
Layer II/III	259 (23.3)	243 (22.6)	148 (16.1)	147 (16.4)
Layer IV	176 (15.8)	164 (15.2)	174 (18.9)	169 (18.9)
Layer V	238 (21.4)	232 (21.5)	200 (21.8)	196 (21.9)
Layer VI	434 (39.2)	436 (40.55)	395 (43.1)	382 (42.7)
Total	1107	1075	917	894
wM1 cortex				
Layer II/III	133 (26.3)	136 (26.9)	97 (23.0)	93 (21.9)
Layer V	226 (44.6)	220 (43.5)	210 (49.6)	212 (49.7)
Layer VI	148 (29.1)	150 (29.6)	116 (27.4)	121 (28.4)
Total	507	505	423	426


Ctl; Control, WD; Whisker-deprived, Hypo; Hypothyroid, wS1; Whisker part of somatosensory cortex, and wM1; Whisker part of primary motor cortices.

#### wM1 cortex 

NADPH-d neurons were labeled in layers II/III, V and VI of the wM1 cortical area in all groups. It should be mentioned that the motor cortex has no granular layer IV and, therefore, is qualified as the agranular cortex. In the Hypo rats, the numbers of NADPH-d positive neurons observed in layers II/III and VI were notably decreased (P<0.05) compared to the Ctl group. Layer V of the wM1 cortex demonstrated no significant differences in nitrergic neurons between rats in the Ctl and Hypo groups. No significant differences were observed between the numbers of NADPH-d positive neurons in WD rats (the Ctl+WD and Hypo+WD groups) and their homologues controls (Fig .3D). The total distribution of nitrergic neurons was 25, 45 and 30% in layers II/III, V and VI (respectively) of the wM1 cortex in the normal rats (Ctl and Ctl+WD groups). As compared with the normal rats, distribution of NADPH-d positive neurons in the CH groups (Hypo and Hypo+WD groups) was 22, 50 and 28% in layers II/III, V and VI (respectively) of the wM1 cortical area (Table 1). 

### Morphometric features of nitrergic neurons of the

#### wS1 and wM1 cortices

NADPH-d positive neurons of the wS1 (160/group) and wM1 (90/group) cortices were quantified using fourmorphometric parameters (see Materials and Methods). Themean soma diameter (24. 9 ± 1. 1 µm) of NADPH-d positiveneurons in the wM1 cortex of intact rats (Ctl group) wassignificantly less (12%, P<0.05) than neurons in the wS1 cortex (28.2 ± 1.4 µm). There were significant distinctionsbetween the dendritic areas of the wS1 and wM1 areas: neurons in the wM1 had fewer 3rd order processes (8.9 ± 0. 3) than the wS1 cells (11.4 ± 0. 7, P<0.01). Using Shollanalysis, the same reduction was observed in the meannumber of PIs (ΣR1-R10, 3. 3 ± 0. 5 vs. 4. 4 ± 0. 4, P<0.01) and PBPs (ΣR1-R10, 1. 5 ± 0. 1 vs. 1.9 ± 0. 1, P<0.05) in the wM1 compared to the wS1 areas respectively. 

#### Soma diameter

Three nitrergic neuronal types were distinguished in the wS1 and wM1 cortical areas with regard to cell soma diameter. Based on our quantitative analysis, these neurons were divided into three types of cell; small (1525 µm), medium (25-35 µm), and large (35-50 µm).

#### wS1 cortex

In the wS1 cortex of both the Hypo and Ctl groups small NADPH-d positive neurons represented about 45% of the total sample. Whisker deprivation increased the number of small nitrergic stained cells in the normal and congenital hypothyroidism conditions by about 16%. Medium nitrergic neurons comprised approximately 40% of the sampled nitrergic neurons of the wS1 cortex in both Hypo and Ctl groups. A 12% decrease was observed following whisker deprivation in both normal and CH rats. The third type of nitrergic neurons of wS1 cortex consisted of large cells, representing about 15% of all those sampled in both Ctl and CH rats. Following whisker deprivation in the normal and CH rats, a 3 % decrease was demonstrated in wS1 cortical area (Table 2). 

#### wM1 cortex

Approximately 64% of the total number of nitrergic neurons sampled from the wM1 cortex in the Hypo and Ctl groups were small. Similar to the wS1 cortex, whisker deprivation increased the proportion of small nitrergic stained cells in the wM1 in both the normal and CH rats by about 13-14%. About 24% of nitrergic neurons sampled from the wS1 cortex in both the Hypo and Ctl groups were medium nitrergic neurons. Following whisker deprivation, a decrease of about 10% was observed in both the normal and CH rats. Large nitrergic neurons represented 12% of all those sampled in the wM1 cortex of the normal and CH rats, and a 3% decrease was observed following whisker deprivation (Table 2). 

**Table 2:** Number and percentage of randomly selected NADPH-d neurons with different diameter soma in the wS1 cortex and the wM1 cortex of experimental groups


	Group			
	Ctln (%)	Ctl+WDn (%)	Hypon (%)	Hypo+WDn (%)

wS1 cortex				
Small	74 (46.2)	100 (62.5)	74 (46.2)	102 (63.7)
Medium	65 (40.6)	45 (28.1)	67 (41.8)	38 (23.7)
Large	21 (13.1)	15 (9.3)	19 (11.8)	20 (12.5)
Total	160	160	160	160
wM1 cortex				
Small	57 (63.3)	69 (76.7)	58 (64.4)	71 (78.9)
Medium	21 (23.3)	13 (14.4)	22 (24.4)	12 (13.3)
Large	12 (13.3)	8 (8.9)	10 (11.1)	7 (7.8)
Total	90	90	90	90


160 labeled NADPH-d neurons of the wS1 cortex and 90 labeled NADPH-d neurons of the wM1 cortex.These neurons were divided into three types of cell; small (15-25 μm), medium (25-35 μm), and large (35-50 μm). Ctl; Control, WD; Whisker-deprived, Hypo; Hypothyroid, wS1; Whisker part of somatosensory cortex, and wM1; Whisker part of primary motor cortices.

### The processes 

#### Length of the longest dendrites

In the wS1 and wM1 cortical areas of the Hypo groups there was a significant change in the length of the longest dendrites in the nitrergic neurons compared to the normal groups (P<0.01 and P<0.05, for wS1 and wM1, respectively). However, following whisker deprivation in both the Ctl+WD and Hypo+WD groups, there was the same significant decrease in the length of the longest dendrites in the NADPH-d positive neurons compared to the Ctl and Hypo groups (P<0.01 and P<0.05, respectively, Fig .3E, F).

#### The number of processes per 1^st^, 2^nd^ and 3^rd^ order

**Fig.4:**
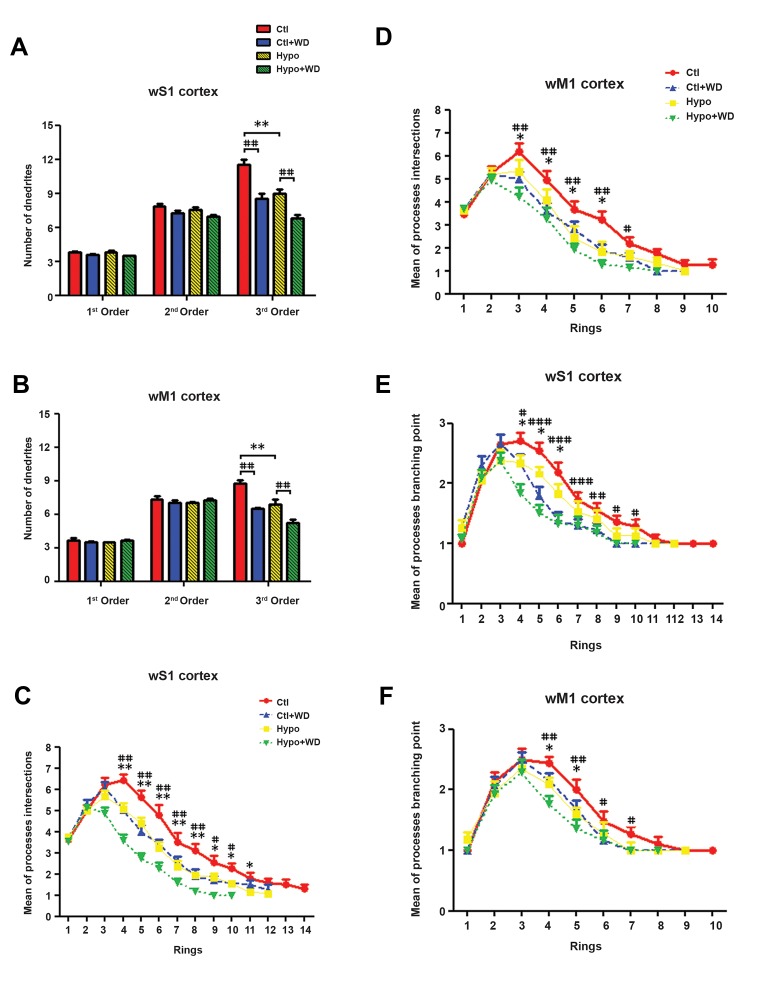
The processes quantification of in the concentric rings for reconstructed NADPH-d labeled neurons of the wS1/M1 cortices in Ctl, Ctl+WD, Hypo, and Hypo+WD rats. A, B. Number of processes per 1st, 2nd and 3rd orders, C, D. Number of process intersections, E, and F. Number of process branching points. All data are expressed as mean ± SEM. *, #; Indicate that all hypothyroid and whisker deprivation values were significantly different from the corresponding controls (*P<0.05, **P<0.01, ***P<0.001) and (#P<0.05, ##P<0.01 and ###P<0.001), Ctl; Control, WD; Whisker-deprived, Hypo; Hypothyroid, wS1; Whisker part of somatosensory cortex, and wM1; Whisker part of primary motor cortices.

The number of processes in the 1^st^, 2^nd^ and 3^rd^ orders of NADPH-d positive cells were counted in the wS1/M1 cortical areas. There was a non-significant difference in the number of 1st and 2nd order processes between nitrergic neurons in the wS1/M1 cortices in the CH and normal rats. However, the number of 3rd order processes in labeled neurons was found to be decreased by 25% in Hypo rats compared to the Ctl group (P<0.01). A similar 25% decrease (P<0.01) in the number of 3rd order processes was observed in the nitrergic neurons of the WD rats (Ctl+WD and Hypo+WD) compared to the corresponding controls (Ctl and Hypo) (Fig .4A, B).

#### The processes intersections 

In the wS1 cortex, there was a significant decrease in the number of processes intersections at concentric rings (4-11) in the Hypo group compared with the Ctl group (CRs 4-8, P<0.01 and CRs 9-11, P<0.05). A similar pattern was observed in the number of PIs of concentric rings (CRs 4-8, P<0.01 and CRs 9-11, P<0.001) in the WD (Ctl+WD and Hypo+WD) groups compared to homologues controls (Ctl and Hypo groups, respectively) (Fig .4C). 

In the wM1, as in the wS1 cortex, NADPH-d positive neurons showed a significant decrease in the number of PIs in the concentric rings in the Hypo group compared with the Ctl group (CRs 3-6, P<0.05). In addition, a significant decrease was observed in the number of PIs in concentric rings (CRs 3-6, P<0.01 and CR7, P<0.05) of the Ctl+WD and Hypo+WD groups compared to the Ctl and Hypo groups, respectively (Fig .4D). 

#### The number of processes branching points

In the wS1 cortex the number of processes branching points (PBPs) in concentric rings (CR4-6, P<0.05) was found to be significantly decreased in the Hypo group compared to the Ctl group (Fig .4E). In addition, the Ctl+WD and Hypo+WD groups showed a significant decrease in the number of PBPs in concentric rings 4-10 (CR4 and CRs 9-10, P<0.05, CR8, P<0.01 and CRs 5-7, P<0.001) compared to the Ctl and Hypo groups, respectively.

In the wM1 cortex the numbers of PBPs in concentric rings (CRs 4-5) were significantly decreased in the Hypo group compared to the Ctl group (P<0.05). The number of PBPs in CRs 4-7. In the Ctl+WD and Hypo+WD groups showed a significant decrease in comparison to the Ctl and Hypo groups, respectively (CRs 4-5, P<0.01 and CR6-7, P<0.05, Fig .4F). 

## Discussion

Similar to our previous studies (4, 27), reduced weight gain was observed in PTU-treated offspring. However, neonatal bilateral whisker trimming did not alter weight gain in the normal and CH rats. These results would appear to be supported by Sun et al. (28) who showed no change in body weight due to bilateral vibrissectomy from postnatal days 2-30 in normal rats. Sullivan et al. (29) observed fast behavioral adaptation in both nipple attachment and huddling behavior in 2 week-old dewhiskered pups. However, da Silva Tenorio et al. (30) reported vibrissae removal to be associated with a slight, but significant, reduction in body weight gain in malnourished pups. 

Differences in the morphometrics of nitrergic neurons were observed across the wS1 and wM1 cortical areas: NADPH-d positive neurons in the wS1 area were greater in size and more branched than NADPH-d positive cortical neurons in the wM1 area. The wS1/M1 cortical nitrergic neurons showed similar morphometric changes in response to whisker deprivation and congenital hypothyroidism interventions. However, the severity of these alterations was less in the wM1 cortex than the wS1 cortex. The heterogeneous morphology of cortical interneurons could reflect a modality-driven specialization in the processing of sensory information (31). 

The results of the present study showed that the relative number and distribution of nitrergic neurons in the wS1/ M1 cortical areas decreased in CH rats while bilateral whisker deprivation does not appear to affect the number or distribution of positively labeled cells. Another previous study also showed that in the olfactory cortex unilateral nares occlusion had little effect on the number of nitrergic cells (32). Moreover, it has been reported that moderate degrees of thyroid hormone insufficiency during the early postnatal period permanently decrease interneuron expression of parvalbumin-positive neurons in the rat hippocampus (33). 

The present study also demonstrated that in normal rats nitrergic neurons in the wS1/M1 cortical areas are located mainly throughout wS1 layers II/III-VI, with a minimum in layer IV and a peak in layer VI. Through the wM1 they are located mainly in layers II-VI with a maximum in layer V. These findings are consistent with previous studies (18, 34). These laminar distributions were mildly altered in hypothyroid rats. The abnormal laminar distribution and drastic decrease in the density of NADPH-d positive neurons could be related, at least in part, to abnormal neuronal migration, cell proliferation or apoptosis of NADPH-d positive neurons during brain development (35).

According to our results there was no difference in the number of nitrergic cell bodies of different sizes (small, medium, and large soma diameter) in the wS1/ M1 cortices in CH rats compared to intact rats. However, a reduction in nitrergic cell bodies (medium and large soma diameter) in the wS1/M1 cortices (were observed in the whisker-trimmed rats compared to the controls. In addition, the results of the present study demonstrate that similar patterns of decreased NADPH-d labeled neurons in the wS1/M1 cortices occur in the processes of nitrergic neurons in both conditions of congenital hypothyroidism and whisker deprivation. Furthermore, the main findings of the present study showed that a long period of sensory deprivation during adolescence has a significant effect on the morphometric modifications of nitrergic neurons of CH rats. Congenital hypothyroidism (35) might modify the connectional phenotype of cortical neurons by altering the relation between laminar fate and connectivity. It has been noted that a lack of correlation between the dendritic trees and their branching complexity with the size of the cell body suggests widespread variance between these parameters (36).

From these findings it could be concluded that both chronic whisker deprivation and congenital thyroid hypofunction could change the pattern of inhibitory neurons in the wS1/M1 cortical circuits. As has been noted, the wS1 cortex receives most of its input from the ventral posterior medial and posterior nuclei of the thalamus through the whiskers to barrel pathway (37). Sensory input from the wM1cortex comes via wS1 (7) and directly from the posterior nucleus of the thalamus (38).

Our observed reduction in cell body size and processes of the nitrergic neurons in the wS1/M1cortical areas are possibly due to decreased thalamocortical inputs in the whisker deprivation groups. These decreases in some of the morphometric properties of nitrergic neurons probably relate directly to reduction in body and brain weight in the hypothyroid groups. However, whisker deprivation induced the same changes in the number, cell body size and processes of NADPH-d positive neurons in the wS1/ M1cortical areas in the normal and hypothyroid rats.

These results suggest that the effects of total whisker deprivation and congenital hypothyroidism on the morphometric characteristics of nitrergic neurons of wS1/ M1 cortical areas are independent. However, further analysis may be required to investigate other physiological aspects of these results in cortical circuits. For example, the study of the dendritic spines of nitrergic neurons by electronic microscope can be useful in this regard. In addition, electrophysiological findings have shown that cortical spreading depression propagation changes have the same pattern in well-nourished rats and vibrissaeremoved malnourished animals (30). However, the relation between biochemical parameters of subclinical protein malnutrition and thyroid homeostasis (39) suggest that thyroid hypofunction may result in an adaptation to malnutrition (40).

## Conclusion

NADPH-d interneurons of the wS1 and wM1cortical areas respond to thyroid hormone deprivation, with similar responses observed in both areas. Differences in morphological characteristics between NADPH-d interneurons in the wS1 and wM1cortical areas may reflect the differences in their functions, as neuronal functions are directly related to the amount of inputs that a neuron can receive. In this regard, brain inhibitory networks in the congenital hypothyroid and whisker deprived rats had shorter and more tortuous branches with reduced number of arbors than those found in the normal rats. This may have important implications for the physiological roles of NADPH-d positive neurons, such as neuronal plasticity, memory formation and regulation of central nervous system blood flow and indicate a way that less ramified NADPH-d interneurons can exert less influence in a specific cortical area.
